# Supplementary material for: Development and piloting of a primary school-based salt reduction programme: Formative work and a process evaluation in rural and urban Malawi
Source: PLOS Glob Public Health. 2023 Aug 30;3(8):e0000867. doi: 10.1371/journal.pgph.0000867 (PMC10468067; doi:10.1371/journal.pgph.0000867)
Supplement: S1 Checklist — (DOCX) [file pgph.0000867.s001.docx]

**Table 5 Consolidated criteria for reporting qualitative studies (COREQ): 32-item checklist**

| No Item | Description |
| --- | --- |
| Domain 1: Research team and reflexivity | |
| *Personal characteristics* | |
| 1. Interviewer/ Facilitator | The facilitators have been mentioned in the acknowledgements (HN, CN) |
| 1. Credentials | First facilitator has a masters in Global Health Policy and the second a diploma in Psychosocial counselling and a certificate in primary teacher training. |
| 1. Occupation | First facilitator was a social scientist and the second a Field supervisor |
| 1. Gender | Both are women |
| 1. Experience and training | They both have over 10 years of qualitative research |
| *Relationship with participants* | |
| 1. Relationship established | Relationship established when the study was introduced to the schools and through the pilot study. |
| 1. Participant knowledge of the interviewer | The participants knew the facilitators in their capacity as researchers. There was no personal connection. |
| 1. Interviewer characteristics | Described above |
| Domain 2: Study design | |
| *Theoretical framework* | |
| 1. Methodological orientation and theory | This is a qualitative study using Normalization Process Theory (NPT) as the underpinning theoretical framework. |
| *Participant selection* | |
| 1. Sampling | The study used purposive sampling |
| 1. Method approach | Used Focus Group Discussions, In-depth interviews and observations. |
| 1. Sample size | Sample size is 96 |
| 1. Non- participation | All participants accepted to participate |
| *Setting* | |
| 1. Setting of data collection | Data collection was done at pilot schools in private spaces |
| 1. Presence of non-participants | All interviews and FGDs only included invited participants and did not include outside parties. |
| 1. Description of sample | The sample included samples of children, parents and teachers of different ages and gender. The children were sampled based on how active in learning they were in class (both ends of the spectrum were included). The teachers were those involved in the delivery of the intervention. The parents were chosen from the class lists. |
| *Data collection* | |
| 1. Interview guide | The study used semi structured interview or FGD guide |
| 1. Repeat interview | There were no repeat interviews |
| 1. Audio/ visual recording | The interviews and FGDs were audio recorded |
| 1. Field notes | The study kept field notes of the interviews FGDs and observations |
| 1. Duration | The interviews/ FGDs lasted 40-60 minutes |
| 1. Data saturation | Data saturation was reached |
| 1. Transcripts returned | No, this was not feasible in this study. |
| Domain 3. Analysis and findings | |
| *Data analysis* | |
| 1. Number of data coders | The data was initially coded by three coders to establish a codebook. The rest of the data was double coded, and coding was discussed in wider coding clinics involving 3 researchers (NP, YC, FSM) |
| 1. Description of the coding tree | This was done described in the data analysis |
| 1. Derivation of themes | The themes were identified from the data |
| 1. Software | Data was analyzed using NVivo 12 |
| 1. Participant checking | In this setting it was not feasible for participant to check the transcripts |
| 1. Quotations presented | Participant quotations have been presented to support the findings of the study. The quotes are identifiable |
| 1. Data and findings consistent | There is consistency between data presented and findings |
| 1. Clarity of major themes | Yes. This is clear in the findings and discussions |
| 1. Clarity of minor themes | Yes. This is clear in the findings and discussions |
|  |  |
