# Supplementary material for: Development and piloting of a primary school-based salt reduction programme: Formative work and a process evaluation in rural and urban Malawi
Source: PLOS Glob Public Health. 2023 Aug 30;3(8):e0000867. doi: 10.1371/journal.pgph.0000867 (PMC10468067; doi:10.1371/journal.pgph.0000867)
Supplement: S1 Text — (DOCX) [file pgph.0000867.s002.docx]

**Interview Guides: Parents, Adolescents, Teachers**

**General perceptions of salt and hypertension education**

1. **Tell me about any information you have received on reducing salt in your diet?**
2. **What do you know about excessive salt intake and health?** **Can you tell me what you have learnt?**
3. **What did you like about the information you received?**
4. **What did you not like about the information you received?**
5. **Was there a specific way you would have liked the information delivered to you?**
6. **What other information about reducing salt would you have liked to hear?**

**Specific perceptions of the Trial:**

1. **What do you think of this trial of an educational intervention in schools?**
2. Does it seem like a good idea or not? Please explain why you think this…
3. Do you think what the children learn in school influence what you do at home?
4. How easy or hard do you think it was it to make the changes in cooking and eating that were being suggested?
5. Can you think of anything that could be changed in the advice given or the way the information is delivered that would be helpful? If yes, explain…..

**Additional Questions For: Primary School Teachers/Head Teachers** *:*

1. **What were your experiences participating in the salt reduction education for primary school going children and their families?**
2. Can you tell me about your experiences participating in this programme? Any challenges?
3. What do you think should be the role of the family to encourage students in this education?
4. What did you think about including parents in the learning?

**Additional Questions for Students and Parents;**

1. What do you think should be the role of the family to encourage students in this education? What did you think about incorporating parents in the learning?
2. Can you tell me about your experiences participating in this programme? Any challenges?
